# Association of Metformin With Pregnancy Outcomes in Women With Polycystic Ovarian Syndrome Undergoing In Vitro Fertilization: A Systematic Review and Meta-analysis

**DOI:** 10.1001/jamanetworkopen.2020.11995

**Published:** 2020-08-03

**Authors:** Yiqing Wu, Mixue Tu, Yun Huang, Yifeng Liu, Dan Zhang

**Affiliations:** 1 Women’s Reproductive Health Research Key Laboratory of Zhejiang Province, Women’s Hospital, Department of Reproductive Endocrinology, Zhejiang University School of Medicine, Hangzhou, Zhejiang, China; 2 Key Laboratory of Reproductive Genetics, Zhejiang University, Ministry of Education, Hangzhou, Zhejiang, China

## Abstract

**Importance:**

Metformin is widely used among women with polycystic ovary syndrome (PCOS). However, its associations with outcomes of in vitro fertilization or intracytoplasmic sperm injection and embryo transfer (IVF/ICSI-ET) in women with PCOS remain controversial.

**Objective:**

To assess whether metformin is associated with improved outcomes of IVF/ICSI-ET in women with PCOS.

**Data Sources:**

PubMed, Embase, and Cochrane were searched from database inception to January 31, 2020.

**Study Selection:**

Only randomized clinical trials (RCTs) were included. Eligible studies enrolled women with PCOS undergoing infertility treatment with IVF/ICSI-ET and reported at least 1 outcome of IVF/ICSI-ET.

**Data Extraction and Synthesis:**

This study followed the Preferred Reporting Items for Systematic Reviews and Meta analyses guidelines. Two authors independently extracted the data. Study quality was evaluated using the GRADE system. Treatment effect was quantified using odds ratios (ORs) with 95% CIs using random-effect models with the Mantel-Haenszel method.

**Main Outcomes and Measures:**

Ovarian hyperstimulation syndrome (OHSS), clinical pregnancy rate, and live birth rate.

**Results:**

A total of 12 RCTs, which collectively included 1123 women with PCOS undergoing infertility treatment with IVF/ICSI-ET, were identified. The risk of OHSS in women randomized to metformin was lower than in women not randomized to metformin (OR, 0.43; 95% CI, 0.24-0.78), although this difference was not significant for women with PCOS with a body mass index of less than 26 (OR, 0.67; 95% CI, 0.30-1.51). There was no significant difference in clinical pregnancy rate (OR, 1.24; 95% CI, 0.82-1.86) or live birth rate (OR, 1.23; 95% CI, 0.74-2.04) in the total population studied. However, in a post hoc analysis among women with a body mass index of 26 or greater, metformin treatment was associated with increased clinical pregnancy rates (OR, 1.71; 95% CI, 1.12-2.60).

**Conclusions and Relevance:**

In this study, metformin treatment was associated with a decreased risk of OHSS but had no association with the overall clinical pregnancy rate or live birth rate among women with PCOS undergoing IVF/ICSI-ET. Metformin treatment should be carefully considered for women with PCOS undergoing IVF/ICSI-ET and may be more preferred for women with a body mass index greater than 26.

## Key Points

**Question:**

Is metformin associated with improved outcomes among women with polycystic ovarian syndrome undergoing in vitro fertilization?

**Findings:**

This meta-analysis of 12 randomized clinical trials, which collectively included 1123 women, found that metformin treatment was associated with a decreased risk of ovarian hyperstimulation syndrome among women with polycystic ovarian syndrome undergoing in vitro fertilization but had no association with clinical pregnancy or live birth rates in the total population studied. However, among women with a body mass index of 26 or greater, metformin treatment was associated with an improved clinical pregnancy rate.

**Meaning:**

The findings of this study suggest that metformin treatment should be carefully considered for women with polycystic ovarian syndrome undergoing in vitro fertilization and may be more preferred among women with a body mass index of 26 or greater.

## Teaser

This systematic review and meta-analysis assesses whether metformin is associated with improved outcomes of in vitro fertilization or intracytoplasmic sperm injection and embryo transfer among women with polycystic ovarian syndrome.

## Introduction

Polycystic ovary syndrome (PCOS), among the most common endocrinopathies associated with reproductive and metabolic disorders, affects 9% to 18% of women.^1^ According to the World Health Organization, PCOS belongs to group II of ovulation disorders and accounts for approximately 80% of women with anovulatory infertility.^1^ Stein and Leventhal^2^ first described PCOS in 1935, but it still presents dilemmas in reproductive medicine.

To date, the most widely accepted diagnostic criteria for PCOS is the Rotterdam criteria (2003), which require that women must meet 2 of the following items: oligo-ovulation or anovulation, clinical and/or biochemical signs of hyperandrogenism, and polycystic ovaries.^3^ Additionally, other well-known disorders characterized by androgen excess should be excluded.

The heterogeneous manifestations of PCOS include infertility, obesity, inappropriate gonadotropin secretion (ie, elevated levels of circulating luteinizing hormone), pregnancy complications, cardiovascular disease, and psychological problems. In addition, metabolic features, especially insulin resistance with accompanying compensatory hyperinsulinemia, are common in women with this disorder.^4,5^ Given that a significant association between testosterone and insulin levels in PCOS has been observed,^6^ it is plausible that insulin resistance could play a role in the pathogenesis of PCOS.

Targeting insulin resistance has improved ovulation and fertility in women with PCOS. During the last decades, this recognition led to many studies regarding the possible role of insulin-sensitizing agents, particularly metformin, in the treatment of PCOS.^6^ Metformin, a biguanide that lowers blood glucose levels in individuals with hyperglycemia and type 2 diabetes, is widely used among women with PCOS. The main pharmacologic action of metformin is to reduce the absorption of glucose from the gastrointestinal tract, inhibit the production of hepatic glucose, and increase insulin-stimulated glucose uptake in the periphery,^6,7^ but the underlying mechanisms of these actions remain unclear.

In the 1990s, a series of studies involving relatively small head-to-head trials (many uncontrolled) indicated that metformin could reduce insulin resistance, increase ovulation, and improve outcomes among women with PCOS undergoing assisted reproductive technology (ART) cycles without, or sometimes with, clomiphene citrate or gonadotrophins.^6,7,8,9,10,11^ Nevertheless, the exact role of metformin in the management of PCOS is still controversial.

Several randomized clinical trials (RCTs) demonstrated that metformin was associated with a significantly higher ovulation rate compared with placebo,^12,13^ but other studies have shown the opposite result.^14^ Moreover, multiple RCTs assessed the role of metformin in clinical pregnancy, live birth rates, and first trimester spontaneous abortion rates and observed the risk of ovarian hyperstimulation syndrome (OHSS). Similarly, many studies showed improved clinical pregnancy and live birth rates,^11,15,16^ improved rates of miscarriage and implantation, and reduced risk of OHSS.^4,8^ However, other studies showed no association of metformin with rates of pregnancy, live birth, and miscarriage.^8,16^

Despite the multitude of RCTs conducted to date, high-quality RCTs designed to answer the specific question of the comparative efficacy of metformin patients with PCOS and with or without obesity are still lacking. Some limited studies have found that body mass index (BMI; calculated as weight in kilograms divided by height in meters squared) may affect the efficacy of metformin.^17,18^ Moreover, the latest guidelines point out that more adequately powered RCTs are needed to carefully define which individuals would benefit from using metformin as well as its suitable dosage and duration of use.^6^

Given the fact that previous studies were insufficient to evaluate the efficacy of metformin in women with PCOS, the present study aimed to systematically review the literature and perform a meta-analysis to clarify whether metformin is associated with improved outcomes in women with PCOS undergoing in vitro fertilization or intracytoplasmic sperm injection and embryo transfer (IVF/ICSI-ET) cycles.

## Methods

We conducted an updated meta-analysis of RCTs to assess the association of metformin treatment during the course of infertility treatment with ART. This study followed the Preferred Reporting Items for Systematic Reviews and Meta-analyses (PRISMA) reporting guideline.^19^

### Search Strategy

We searched PubMed, Embase, and Cochrane databases from database inception to January 31, 2020. The search strategy used the format of participants, interventions, comparisons, outcomes, and study design (PICOS).^19^ All articles were limited to English language involving human participants. We applied a combination of Medical Subject Headings and free-text terms, including their variants, to search in PubMed and Cochrane library. We used the PICOS search in Embase. The search terms were *polycystic ovary syndrome*, *metformin*, *randomized controlled trial*, and *reproductive techniques, assisted*. The detailed search strategies are shown in the eAppendix in the Supplement.

### Study Selection and Data Extraction

Two of us (Y.W. and M.T.) independently screened the titles and abstracts of all articles and excluded irrelevant articles. According to the exclusion and inclusion criteria, 2 of us (Y.W. and M.T.) independently read the full text of all relevant articles and retrieved the eligible studies. Any discrepancies were discussed by the 2 of us and addressed by the third reviewer (Y.L.). Inclusion criteria were as follows: participants had PCOS diagnosis according to the Rotterdam criteria or other standard diagnostic criteria; participants were aged 20 to 45 years; participants used metformin as 1 intervention; participants underwent IVF/ICSI-ET; study was an RCT. The exclusion criteria were as follows: participants had a history of diabetes or other endocrinological disease; participants used metformin before study; data missing or lost to follow-up; study not reported in English; RCT did not have a parallel controlled design. Data were extracted from included studies by 2 of us (Y.W. and Y.H.). The features of these studies that potentially related to the outcomes were extracted as follows: first author, publication year, location, participant characteristics, protocol of control ovarian stimulation, total number of women in the intervention and control groups, and other baseline characteristics. The outcome measures were risk of OHSS, clinical pregnancy rate, live birth rate, and miscarriage rate. We defined OHSS based on patient reports of OHSS symptoms requiring outpatient follow-up or hospitalization, coasting performed, or IVF/ICSI-ET cancelled to avoid development of OHSS. The clinical pregnancy rate was defined as the total number of cases with at least 1 sac on ultrasound divided by the total number of initiated cycles. The live birth rate was defined as the total number of cases with at least 1 baby born after 28 weeks of gestation divided by the total number of initiated cycles. The miscarriage rate was defined as the total number of cases with at least 1 clinical pregnancy that was subsequently spontaneously miscarried divided by the total number of initiated cycles.

### Statistical Analysis

We combined data and used odds ratios (ORs) with 95% CIs to estimate treatment effects using random-effect models with the Mantel-Haenszel method in the software Review Manager version 5 (Cochrane Collaboration). Heterogeneity across the studies was assessed with the *I*^2^ statistic, with *I*^2^ greater than 50% indicating substantial heterogeneity. We considered the comparison a significant difference if *P* < .05, and all tests were 2-tailed. We conducted a post hoc subgroup analysis by dividing into 2 groups based on BMI (<26 and ≥26) and addressed the sensitivity analysis with the funnel plot.

We intended to use the funnel plot to explore publication bias. Risks of bias were assessed by 2 of us (Y.W. and M.T.) using the Cochrane Handbook methods.^20^ Every study was designated as having a low, high, or unclear risk of various biases and then pooled in a summary graph of bias. We used the software GRADEpro^21^ to evaluate the methodological quality of included studies and synthesized a summary of outcomes table that revealed the evidence quality (ie, high, moderate, or low) of each outcome.

## Results

### Description of Studies

#### Search Results

**Figure 1.**
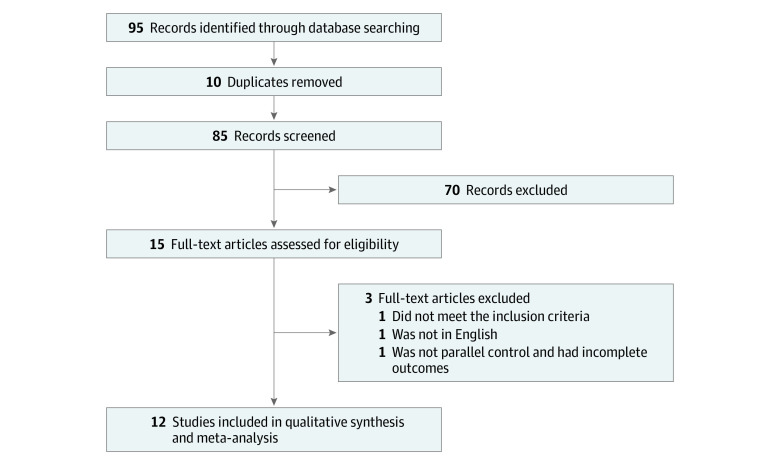
Flow Diagram of Study Selection

The flow of the selection process is shown in Figure 1. Our meta-analysis retrieved 95 articles. By removing duplicates and screening the title and abstract, only 15 articles were includeand.^14,16,22,23,24,25,26,27,28,29,30,31,32,33,34^ After full-text assessment, 1 article was excluded because participants did not meet the inclusion criteria, 1 article was excluded because it did not have a parallel control and had incomplete outcomes, and 1 article was excluded because it did not report findings in English (eTable 3 in the Supplement). Finally, 12 RCTs^14,16,22,23,24,25,26,27,28,29,30,31^ were included in the meta-analysis. In total, 1123 women with PCOS were randomized, with 564 (50.2%) to metformin and 559 (49.8%) to control.

#### Baseline Characteristics

Ten studies^14,16,22,23,24,25,26,27,28,29^ used the Rotterdam criteria for diagnosis of PCOS. Eleven studies^14,16,22,23,24,25,26,27,28,30,31^ provided full baseline characteristics of the participants in both groups (ie, site, age, BMI, and control ovarian stimulation protocol). One study^29^ did not provide any baseline characteristics for participants. Main characteristics and specific interventions of included studies are presented in eTable 1 and eTable 2 in the Supplement. Eight studies^14,16,22,24,25,26,28,31^ reported the full outcomes (ie, OHSS rate, clinical pregnancy rate, and live birth rate). One study^30^ did not report OHSS incidence; 1 study^29^ did not report clinical pregnancy rates; and 4 studies^23,27,29,30^ did not report live birth rates. Only 4 studies^14,16,27,30^ reported miscarriage rates.

#### Quality and Risk of Studies

**Figure 2.**
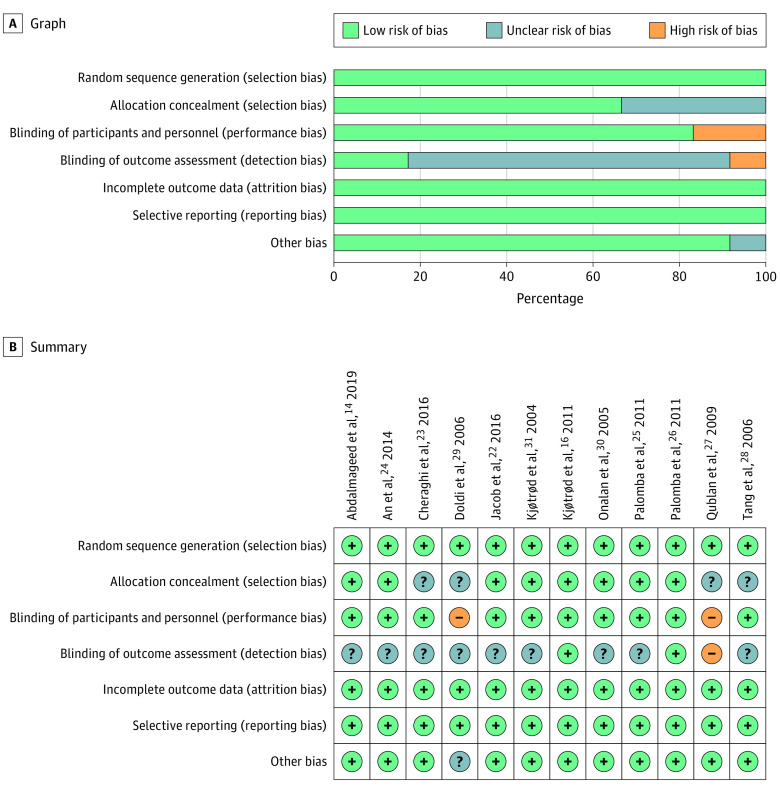
Risk of Bias Graph B, Green plus signs indicate low risk of bias; gray question marks, unclear risk of bias; and orange minus signs, high risk of bias.

The quality of studies was evaluated by GRADEpro, as shown in the Table. The risk of bias was assessed by Cochrane Handbook, as shown in Figure 2.

**Table.** Summary of Outcomes

Outcome	Assumed risk among control group study participants per 1000 patients^a^	Assumed moderate risk among control group per 1000 patients^a^	Corresponding risk among metformin group study participants per 1000 patients (95% CI)^a^	Corresponding moderate risk among metformin group per 1000 patients (95% CI)^a^	Relative effect, OR (95% CI)	Participants, No. (studies, No.)	Quality of evidence^b^
Risk of OHSS							
All participants	179	125	86 (52-137)	58 (34-94)	0.43 (0.25-0.73)	947 (11)	High
Participants with BMI ≥26	167	165	48 (24-93)	47 (23-92)	0.25 (0.12-0.51)	482 (6)	High
Participants with BMI <26	200	147	140 (72-259)	101 (51-194)	0.65 (0.31-1.40)	425 (4)	High
Clinical pregnancy rate							
All participants	335	304	385 (293-484)	351 (264-448)	1.24 (0.82-1.86)	1015 (11)	Moderate^c^
Participants with BMI ≥26	280	219	400 (304-503)	324 (239-422)	1.71 (1.12-2.60)	482 (6)	High
Participants with BMI <26	385	364	305 (235-484)	330 (219-462)	0.86 (0.49-1.50)	533 (5)	Moderate^c^
Live birth rate							
All participants	303	291	349 (244-471)	335 (233-456)	1.23 (0.74-2.04)	811 (8)	Moderate^c^
Participants with BMI ≥26	276	260	371 (268-487)	353 (252-467)	1.55 (0.96-2.49)	386 (4)	High
Participants with BMI <26	329	291	322 (170-523)	285 (147-479)	0.97 (0.42-2.24)	425 (4)	Moderate^c^

Abbreviation: BMI, body mass index (calculated as weight in kilograms divided by height in meters squared); OR, odds ratio.

^a^ The assumed risk is the median control group risk across studies. The corresponding risk and its 95% CI are based on the assumed risk in the control group and the relative effect (ie, OR) and its 95% CI.

^b^ Quality of evidence was based on the GRADE Working Group grades of evidence, as follows: high, further research is very unlikely to change our confidence in the estimate of effect; moderate, further research is likely to have an important effect on our confidence in the estimate of effect and may change the estimate; low, further research is very likely to have an important effect on our confidence in the estimate of effect and is likely to change the estimate; very low, we are very uncertain about the estimate.

^c^ Heterogeneity was high, with *I*^2^ greater than 50%.

### Outcomes

#### OHSS Rate

**Figure 3.**
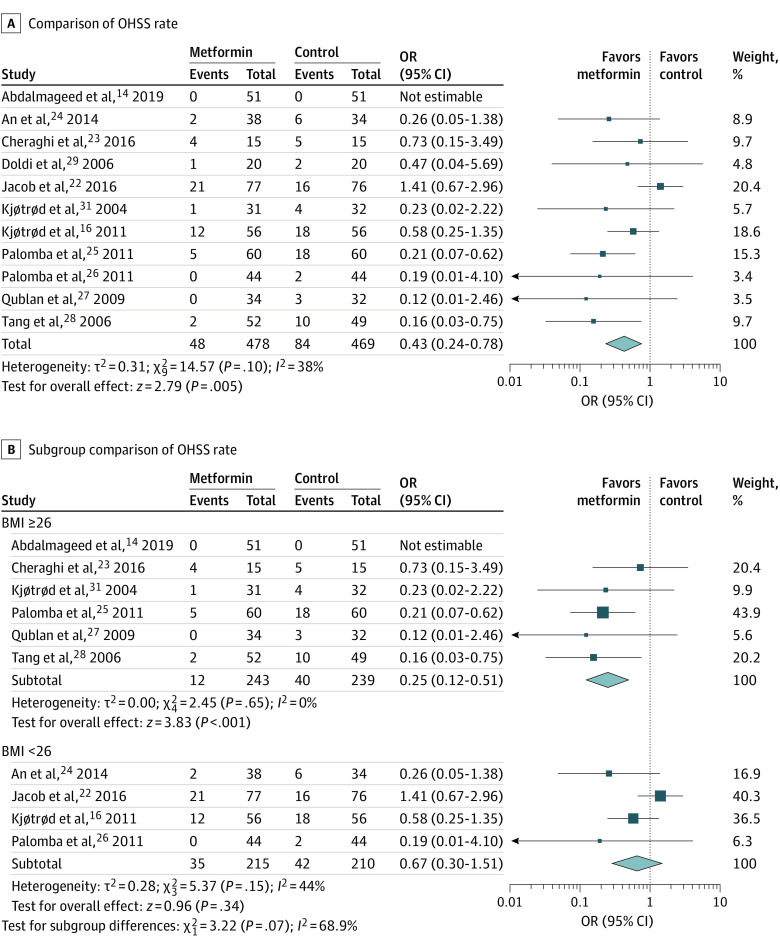
Association of Metformin With Ovarian Hyperstimulation Syndrome (OHSS) Rate BMI indicates body mass index (calculated as weight in kilograms divided by height in meters squared); OR, odds ratio.

Eleven studies^14,16,22,23,24,25,26,27,28,29,31^ reported the OHSS rate, and 1 study^30^ reported the incidence of OHSS as 0 in both metformin and control groups. In the studies included in the analysis, 10 studies^14,16,23,24,25,26,27,28,30,31^ used long gonadotropin-releasing hormone–agonist stimulation protocol, and the others^22,29^ used gonadotropin-releasing hormone–antagonist protocol. Women in the metformin group had lowers odds of OHSS than women in the control group (11 RCTs; 947 participants; OR, 0.43; 95% CI, 0.24-0.78; *I*^2^ = 38%; *P* = .005) (Figure 3A).

In addition, we divided studies into 2 groups based on BMI (ie, <26 and ≥26). In the subgroup with BMI of 26 or greater, women in the metformin group had lower odds of OHSS than women in the control group (6 RCTs^14,23,26,27,28,31^; 482 participants; OR, 0.25; 95% CI, 0.12-0.51; *I*^2^ = 0%; *P* < .001). However, no difference in OHSS rate was observed in the subgroup with BMI of less than 26 (4 RCTs^16,22,24,26^; 425 participants; OR, 0.67; 95% CI, 0.30-1.51; *I*^2^ = 44%; *P* = .34) (Figure 3B).

#### Clinical Pregnancy Rate

**Figure 4.**
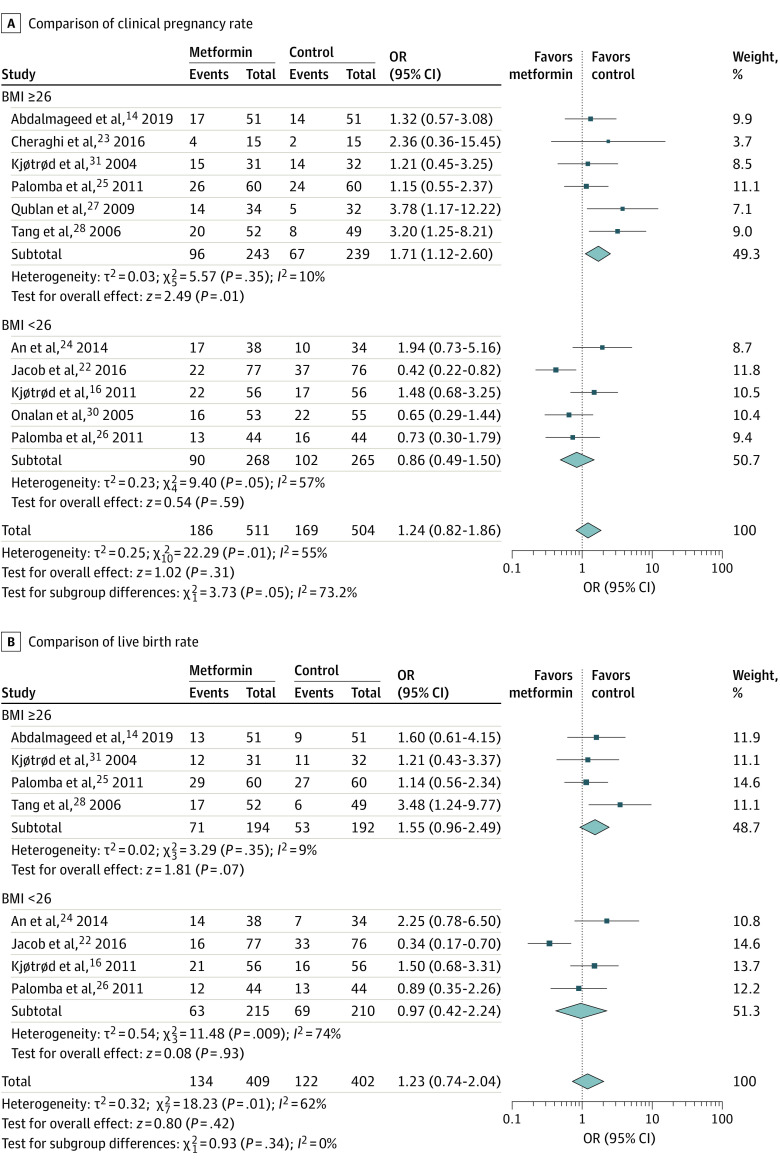
Association of Metformin With Clinical Pregnancy and Live Birth Rates BMI indicates body mass index (calculated as weight in kilograms divided by height in meters squared); OR, odds ratio.

Eleven studies^14,16,22,23,24,25,26,27,28,30,31^ were pooled when analyzing the outcome of clinical pregnancy rate. Metformin was not associated with the clinical pregnancy rate (11 RCTs^14,16,22,23,24,25,26,27,28,30,31^; 1015 participants; OR, 1.24; 95% CI, 0.82-1.86; *I*^2^ = 55%; *P* = .31) (Figure 4A). Because of statistical heterogeneity, we similarly addressed subgroup analysis according to BMI. We observed that heterogeneity was reduced, and there was a significant difference in clinical pregnancy rate (6 RCTs^14,23,25,27,28,31^; 482 participants; OR, 1.71; 95% CI, 1.12-2.60; *I*^2^ = 10%; *P* = .01) in the subgroup of women with BMI of 26 or greater (Figure 4A).

#### Live Birth Rate

This comparison included 8 studies.^14,16,22,24,25,26,28,31^ There was no evidence of a difference in live birth rate between the metformin and control groups (8 RCTs^14,16,22,24,25,26,28,31^; 811 women; OR, 1.23; 95% CI, 0.74-2.04; *I*^2^ = 62%; *P* = .42) (Figure 4B). Because of statistical heterogeneity, we conducted the subgroup analysis based on BMI. The comparison showed no significant difference in live birth rate between the metformin and control groups in women with BMI of 26 or greater (4 RCTs^14,25,28,31^; 386 women; OR, 1.55; 95% CI, 0.96-2.49; *I*^2^ = 9%; *P* = .07). Similar results were found in the other subgroup (4 RCTs^16,22,24,26^; 425 women; metformin group vs control group: OR, 0.97; 95% CI, 0.42-2.24; *I*^2^ = 74%; *P* = .93) (Figure 4B).

#### Miscarriage Rate

This outcome included 4 studies.^14,16,27,30^ There was no evidence of a difference in miscarriage rate between the metformin and control groups (4 RCTs^14,16,27,30^; 388 women; OR, 0.58; 95% CI, 0.24-1.39; *I*^2^ = 0%; *P* = .22) (eFigure 1 in the Supplement).

### Sensitivity Analyses

The funnel plot (eFigure 2 in the Supplement) did not reveal distinct publication bias in this meta-analysis. We conducted the subgroup comparisons to reduce statistical heterogeneity. In addition, we tried to perform a subgroup analysis based on the duration of metformin treatment, but there were no substantial changes in the outcomes.

## Discussion

Results from this meta-analysis showed that metformin was associated with a decrease in the rate of OHSS in women with PCOS undergoing IVF/ICSI-ET. The potential mechanisms of the association are still unclear. Metformin may reduce the rate of OHSS because of the down-expression of vascular endothelial growth factor,^35^ the most important factor in the pathophysiology of OHSS. Other explanations have also been proposed. Some studies indicate that metformin could function as a hyperinsulinemia inhibitor or could decrease the level of estradiol on the trigger day.^11^ Our results showed no heterogeneity and high-quality evidence, suggesting that women with PCOS undergoing IVF/ICSI-ET facing high risk of OHSS should consider metformin cotreatment.

Metformin is widely used during pregnancy but with regional difference. Metformin coadministration before and during an ART cycle for women with PCOS^36^ has been part of protocol in many ART clinics. Recent studies have reported that metformin can be transported to child from mother through placental circulation during pregnancy^37^ and that its concentration in fetal cord blood is close to that of maternal blood. Moreover, metformin treatment does not lower the risk of developing gestational diabetes in high-risk women.^38^ Because of the physiological role of metformin in anticell growth and proapoptosis, metformin treatment should be carefully considered during pregnancy.

Our meta-analysis found that there was no association between clinical pregnancy or live birth rates and use of metformin in women with PCOS undergoing IVF/ICSI-ET. However, when we divided participants into 2 subgroups according to BMI, we found that metformin was associated with a significant increase in clinical pregnancy rates among women with PCOS and a BMI of 26 or greater. The underlying mechanism of this result is also unclear. Recent research has found that metformin might reverse the impaired glycolysis directly and normalize the mitochondrial function in women with PCOS and endometrial hyperplasia,^39^ which further improves the fertilization process and increases the clinical pregnancy rate. Women with PCOS are more likely to have obesity and a higher BMI. Another meta-analysis^40^ summarized 47 studies and concluded that metformin could lower triglyceride levels in patients with PCOS who did not have diabetes. The mechanism of metformin regulating lipid metabolism was possibly through improving oxidative stress status.^41^ Women with a BMI of 26 or greater might have more body fat and could benefit more from metformin treatment than women with lower BMI.

In a subgroup analysis based on the duration of metformin treatment, there were no substantial changes in the outcomes. One guideline^6^ pointed out that stopping metformin treatment at the initiation of gestation did not influence the live birth rate. However, 1 RCT^42^ reported that metformin might reduce the incidence of late miscarriage and preterm birth when the treatment duration was sustained from the late first trimester to delivery.

### Limitations

This study has limitations. This meta-analysis was neither the first nor the only study to examine metformin cotreatment in patients with PCOS undergoing IVF/ICSI-ET. However, we updated the analysis to include newly published articles, provide greater detail on pregnancy outcomes, and focus on subgroup analysis according to BMI. In addition, the included studies did not have all outcomes or baseline characteristics that we needed. Lack of outcome data limited the number of participants and affected our results. Moreover, among the included studies, most participants were of European descent, which may affect generalizability to other racial and ethnic groups. More studies are needed to make the results more applicable to people in other regions, such as Asia. Furthermore, a comment on 2 follow-up RCTs^43^ about metformin increasing the risk of overweight in early childhood suggests that intervention studies with metformin need to pay attention to the long-term health outcomes of the offspring. In this meta-analysis, the included RCTs only provided data up to the live birth rate, and we were therefore unable to provide information about safety of the offspring.

## Conclusions

This meta-analysis showed metformin treatment was associated with a decreased risk of OHSS but had no association with the overall clinical pregnancy rate or live birth rate among women with PCOS undergoing IVF/ICSI-ET. For women with a BMI of 26 or greater, metformin treatment was associated with an increased clinical pregnancy rate. Metformin treatment should be carefully considered for women with PCOS undergoing IVF/ICSI-ET and may be more preferred among women with a BMI of 26 or greater.

## Notes

Supplement. **eAppendix. **Supplementary Methods **eFigure 1. **Forest Plot of Comparison of Miscarriage Rate **eFigure 2. **Funnel Graph of Comparison of the Outcomes of Clinical Pregnancy Rate **eTable 1.** Baseline Characteristics of Included Studies **eTable 2.** Specific Interventions of Included Studies **eTable 3.** Excluded Studies
