# Supplementary material for: Association of Metformin With Pregnancy Outcomes in Women With Polycystic Ovarian Syndrome Undergoing In Vitro Fertilization: A Systematic Review and Meta-analysis
Source: JAMA Netw Open. 2020 Aug 3;3(8):e2011995. doi: 10.1001/jamanetworkopen.2020.11995 (PMC7399751; doi:10.1001/jamanetworkopen.2020.11995)
Supplement: Supplement. — eAppendix. Supplementary Methods eFigure 1. Forest Plot of Comparison of Miscarriage Rate eFigure 2. Funnel Graph of Comparison of the Outcomes of Clinical Pregnancy Rate eTable 1. Baseline Characteristics of Included Studies eTable 2. Specific Interventions of Included Studies eTable 3. Excluded Studies [file jamanetwopen-3-e2011995-s001.pdf]

Supplementary Online Content

Wu Y, Tu M, Huang Y, Liu Y, Zhang D. Association of metformin with pregnancy outcomes in women with polycystic ovarian syndrome undergoing in vitro fertilization: a systematic review and meta-analysis. *JAMA Netw Open*. 2020;3(8):e2011995. doi:10.1001/jamanetworkopen.2020.11995

eAppendix. Supplementary Methods
eFigure 1. Forest Plot of Comparison of Miscarriage Rate
eFigure 2. Funnel Graph of Comparison of the Outcomes of Clinical Pregnancy Rate
eTable 1. Baseline Characteristics of Included Studies
eTable 2. Specific Interventions of Included Studies
eTable 3. Excluded Studies

This supplementary material has been provided by the authors to give readers additional information about their work.

© 2020 Wu Y et al. *JAMA Network Open.*

eAppendix. Supplementary Methods

1. PubMed search：

((((((“Dimethylbiguanidine” OR “Dimethylguanylguanidine” OR “Glucophage” OR “Metformin Hydrochloride” OR “Hydrochloride, Metformin” OR “Metformin HCl” OR “HCl, Metformin”)) OR "Metformin"[Mesh])) AND ((("Assisted Reproductive Technique" OR "Reproductive Technique, Assisted" OR "Technique, Assisted Reproductive" OR "Techniques, Assisted Reproductive" OR "Assisted Reproductive Technics" OR "Assisted Reproductive Technic" OR "Reproductive Technic, Assisted" OR "Reproductive Technics, Assisted" OR "Technic, Assisted Reproductive" OR "Technics, Assisted Reproductive" OR "Assisted Reproductive Techniques" OR "Reproductive Technology, Assisted" OR "Assisted Reproductive Technologies" OR "Assisted Reproductive Technology" OR "Reproductive Technologies, Assisted" OR "Technologies, Assisted Reproductive" OR “Technology, Assisted Reproductive”)) OR "Reproductive Techniques, Assisted"[Mesh])) AND ((("Clinical Trials, Randomized" OR "Trials, Randomized Clinical" OR "Controlled Clinical Trials, Randomized")) OR "Randomized Controlled Trial" [Publication Type])) AND ((("Ovary Syndrome, Polycystic" OR "Syndrome, Polycystic Ovary" OR "Stein-Leventhal Syndrome" OR "Stein Leventhal Syndrome" OR "Syndrome, Stein-Leventhal" OR "Sclerocystic Ovarian Degeneration" OR "Ovarian Degeneration, Sclerocystic" OR "Sclerocystic Ovary Syndrome" OR "Polycystic Ovarian Syndrome" OR "Ovarian Syndrome, Polycystic" OR "Polycystic Ovary Syndrome 1" OR "Sclerocystic Ovaries" OR "Ovary, Sclerocystic" OR "Sclerocystic Ovary")) OR "Polycystic Ovary Syndrome"[Mesh])

2. Embase search

'ovary polycystic disease'/exp AND 'metformin'/exp AND 'randomized controlled trial'/exp AND 'ovary hyperstimulation'/exp

3. Cochrane search

#1 MeSH descriptor: [Polycystic Ovary Syndrome] explode all trees

#2 ("polycystic ovary syndrome"):ti,ab,kw (Word variations have been searched)

#3 MeSH descriptor: [Metformin] explode all trees

#4 (metformin):ti,ab,kw (Word variations have been searched)

#5 MeSH descriptor: [Randomized Controlled Trial] explode all trees

#6 ("Randomized controlled trial"):ti,ab,kw (Word variations have been searched)

© 2020 Wu Y et al. *JAMA Network Open.*

#7 MeSH descriptor: [Reproductive Techniques, Assisted] explode all trees

#8 ("Assisted Reproductive Techniques"):ti,ab,kw (Word variations have been searched)

#9 (#1 OR #2) AND (#3 OR #4) AND (#5 OR #6) AND (#7 OR #8)

© 2020 Wu Y et al. *JAMA Network Open.*

eFigure1. Forest Plot of Comparison of Miscarriage Rate

Study or Subgroup	Metformin Events	Metformin Total	Control Events	Control Total	Weight	Odds Ratio M-H, Random, 95% CI
Abdalmageed 2018	4	51	5	51	40.7%	0.78 [0.20, 3.10]
Kjøtrød 2011	1	56	4	56	15.6%	0.24 [0.03, 2.18]
Onalan 2005	1	34	4	32	15.3%	0.21 [0.02, 2.01]
Qublan 2009	3	53	3	55	28.4%	1.04 [0.20, 5.40]
**Total (95% CI)**		**194**		**194**	**100.0%**	**0.58 [0.24, 1.39]**
Total events	9		16			

Heterogeneity: Tau² = 0.00; Chi² = 2.08, df = 3 (P = 0.56); I² = 0%
Test for overall effect: Z = 1.23 (P = 0.22)

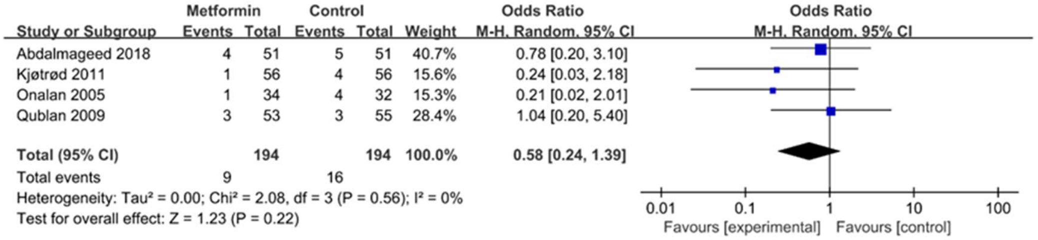

© 2020 Wu Y et al. *JAMA Network Open.*

eFigure2. Funnel Graph of Comparison of the Outcomes of Clinical Pregnancy Rate

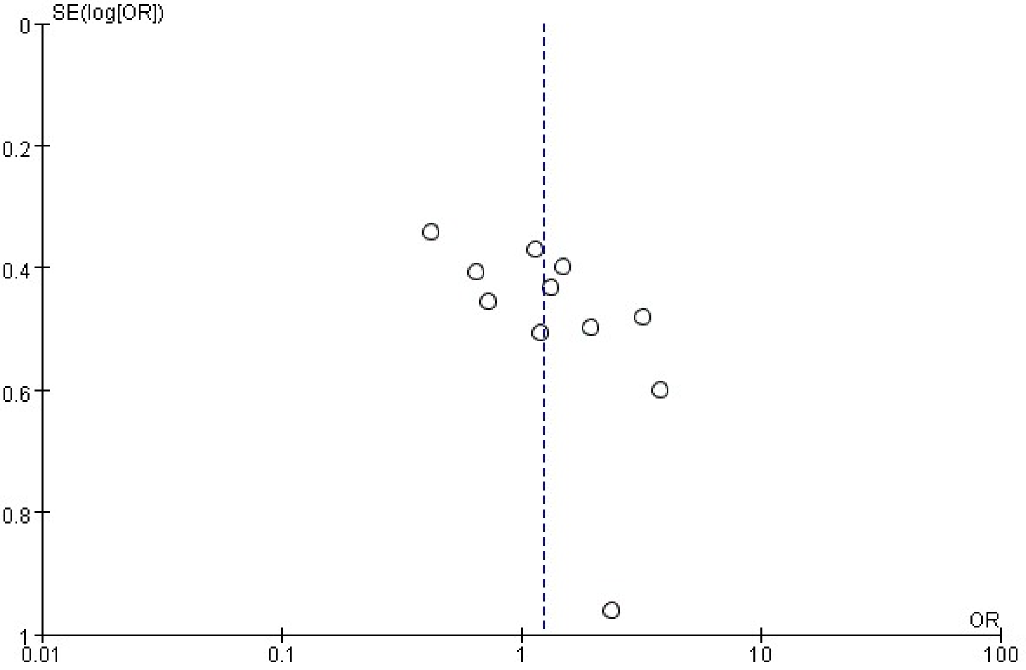

© 2020 Wu Y et al. *JAMA Network Open.*

eTable 1. Baseline Characteristics of Included Studies

Author	Year	Site	PCOS Diagnosis	COS Protocol	Number Metformin	Number Control	Age(year) Metformin	Age(year) control	BMI(kg/m^2) Metformin	BMI(kg/m^2) Control
Abdalmageed	2018	Egypt	Rotterdam criteria	GnRH agonist	51	51	31.1(3.7)	31.9(3.6)	33.8(4.4)	32.9(6.7)
Jacob	2016	UK	Rotterdam criteria	GnRH antagonist	77	76	29.9(4.4)	29.6(3.9)	25.3(3.4)	25(3.3)
Cheraghi	2014	Iran	Rotterdam criteria	GnRH agonist	15	15	28.1(3.4)	27.9(2.8)	27.9(3.1	26.9(2.3)
An	2013	China	Rotterdam criteria	GnRH agonist	41	43	28.7(4.2)	28.4(4)	24(3)	24.2(3.2)
Kjøtrød	2011	Nordic	Rotterdam criteria	GnRH agonist	74	75	29.6(3.4)	29.5(3.8)	24(2.7)	23.6(2.8)
Palomba	2011	Italy	Rotterdam criteria	GnRH agonist	60	60	28.5(3)	29(3)	26.5(2.8)	27(2.7)
Palomba2	2011	Italy	Rotterdam criteria	GnRH agonist	44	44	40(2.8)	39(3.3)	25.4(2.8)	26(2.9)
Qublan	2009	Jordan	Rotterdam criteria	GnRH agonist	34	32	34.6(4.3)	33.8(3.9)	32.2(3)	31.9(3)
Tang	2006	UK	Rotterdam criteria	GnRH agonist	52	49	31.1(4)	31.1(4)	27.9(5.6)	26.9(4.8)
Doldi	2006	Italy	Rotterdam criteria	GnRH antagonist	20	20	/	/	/	/
Onalan	2005	Turkey	NIH consensus	GnRH agonist	53	55	29.3(3.9)	29.8(5.3)	25(5.5)	23.5(3.8)

© 2020 Wu Y et al. *JAMA Network Open.*

Kjøtrød	2004	Norway	NIH consensus	GnRH agonist	31	32	30.2(3.7)	28.9(3.6)	28.6(5.0)	29.9(5.8)

PCOS: Polycystic ovary syndrome, GnRH: gonadotropin-releasing hormone. Rotterdam criteria referred the diagnosis of PCOS was following the Rotterdam Consensus. Intervention duration means the duration of metformin or placebo treatment. Baseline characteristics were calculated and represented in: number, mean (SD).

© 2020 Wu Y et al. *JAMA Network Open.*

eTable 2. Specific Interventions of Included Studies

Author	Year	Dose of Intervention	Duration of intervention
Abdalmageed	2018	Metformin 500 mg once daily	Start from the date of controlled ovarian stimulation (COS), and until the day of pregnancy check. If pregnancy test was positive, the patient was instructed to continue metformin treatment during the first 12 weeks of gestation in both groups.
Jacob	2016	Metformin 850 mg twice daily	Start from mid luteal stage or Day 1 of the period, and until the day before egg collection.
Cheraghi	2014	Metformin 500 mg three times daily	Start from the third day of their last menstrual period in the previous cycle, and until the day of oocyte aspiration.
An	2013	Metformin 500 mg three times daily	Start from ⩾12 weeks prior to COS
Kjøtrød	2011	Metformin was gradually increased from 500 to 2000 mg once daily	Start from ⩾12 weeks prior to COS, and until the day of pregnancy testing.
Palomba	2011	Metformin 500 mg three times daily	Start from the day of GnRH-a administration, and until the day of a positive pregnancy test or menstrual bleeding appeared.
Palomba2	2011	Metformin 500 mg three times daily	Start form 1 month before COS until a positive pregnancy test or menstrual bleeding occurred
Qublan	2009	Metformin 850 mg twice daily	Start from 1 month before the IVF treatment, and until the day of pregnancy test. If the test was positive, metformin was continued for first 12 weeks of gestation in the metformin-treated group.
Tang	2006	metformin 850 mg twice daily	Start from the first day of down-regulation, and until the day of egg retrieval
Doldi	2006	Metformin 1500 mg daily	Start from two months before the IVF treatment, and until the day of embryo transfer

© 2020 Wu Y et al. *JAMA Network Open.*

Onalan	2005	Metformin 850 mg twice or three times daily according to body mass index (BMI) <28kg/m^2 or BMI ≥28 kg/m^2	Start form 8 weeks before their first ICSI cycles, and until a positive pregnancy test
Kjøtrød	2004	Metformin 500 mg twice daily	Start form at least 16 weeks, and until the day of ovulation induction with HCG injection.

© 2020 Wu Y et al. *JAMA Network Open.*

eTable 3. Excluded Studies

Author	Year	Rational
Fedorcsák P	2003	This study is a two-phase cross-over study, which doesn’t meet our inclusion criteria.
Visnová H	2003	It is an article in Czech and doesn’t meet our inclusion criteria.
Swanton A	2011	Participates didn’t meet the inclusion criteria. In this article, participates were confirmed to PCO but not PCOS.

© 2020 Wu Y et al. *JAMA Network Open.*
